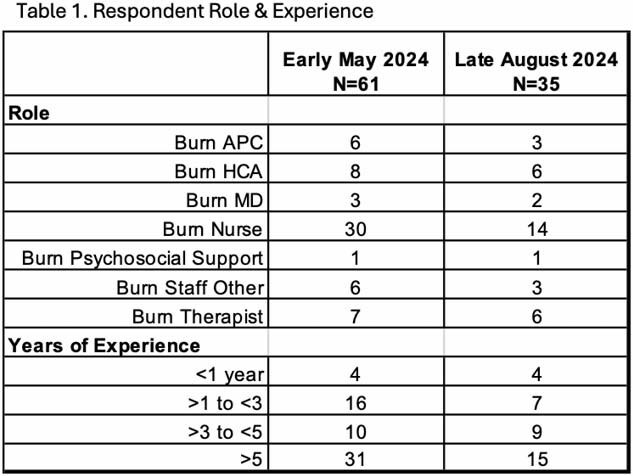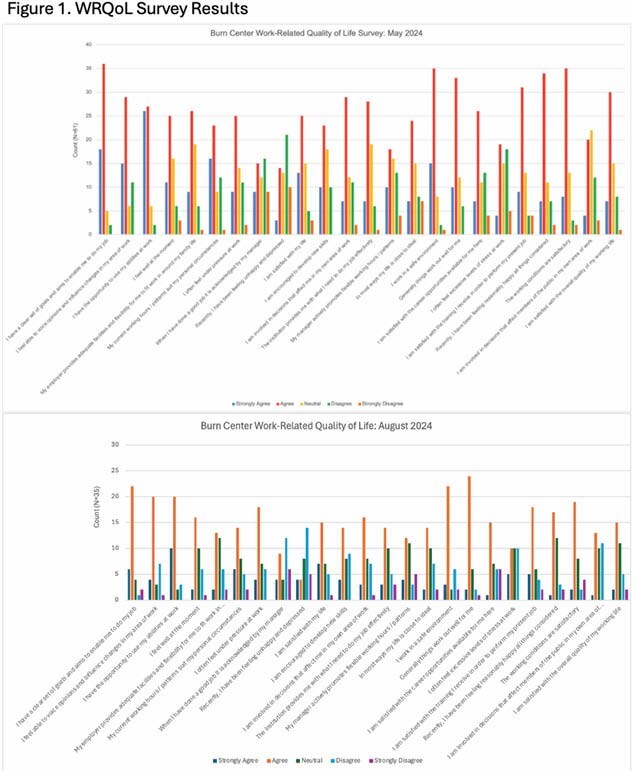# 945 Work-Related Quality of Life in Burn Center Staff

**DOI:** 10.1093/jbcr/iraf019.476

**Published:** 2025-04-01

**Authors:** Emily Beutler, Micayla Kotarski, Callie Thompson

**Affiliations:** University of Utah Health Burn Center; University of Utah Health Burn Center; University of Utah Health Burn Center

## Abstract

**Introduction:**

Achieving optimal outcomes in burn care requires a specialized interdisciplinary team. In recent years, concerns for the short- & long-term stability of the burn workforce have been rising as burnout in the entire healthcare industry has reached all-time highs. To understand our burn center Work-Related Quality of Life (WRQoL) & the factors that may impact it, we initiated a quarterly survey of our Regional Burn Center staff. Here, we describe the novel findings of that survey covering a 6-month (mo.) period.

**Methods:**

After IRB approval, we sent an anonymous email survey to all burn center staff at our ABA-verified Regional Burn Center in May & August 2024. We collected minimal demographic data & inquired about WRQoL, feelings of safety, & intention to leave.

**Results:**

The May survey had a 41% response rate while the August survey had a 25% response rate. Demographics of respondents are in Table 1. The responses to the WRQoL questions for the 2 surveys are in Figure 1. A stable percentage reported that they consider leaving their job at least every few months, 44% & 43%. The percentage reporting a verbal assault at work within the last 3 mo. increased, 35% to 45%. Lastly, 13-20% of respondents reported having an event in the last 3 mo. that they thought about more than they’d like or are still working through.

**Conclusions:**

Our results indicate that our burn center staff are experiencing increasing stress at work & decreasing satisfaction with the quality of their working life over a 6-mo. period. Any discussion regarding the causes of this finding would be hypothetical. Regardless of the reason(s) for these findings, we should all feel an urgency to find interventions to improve WRQoL in our burn center staff as a near-majority regularly think about leaving the job.

**Applicability of Research to Practice:**

Understanding the WRQoL of burn center staff is critical for future interventions to ensure the stability, growth, & continued success of our burn centers.

**Funding for the Study:**

N/A